# SPAK-p38 MAPK signal pathway modulates claudin-18 and barrier function of alveolar epithelium after hyperoxic exposure

**DOI:** 10.1186/s12890-021-01408-7

**Published:** 2021-02-15

**Authors:** Chih-Hao Shen, Jr-Yu Lin, Cheng-Yo Lu, Sung-Sen Yang, Chung-Kan Peng, Kun-Lun Huang

**Affiliations:** 1 Division of Pulmonary and Critical Care Medicine, Department of Internal Medicine, Tri-Service General Hospital, National Defense Medical Center, No. 325, Section 2, Cheng-Gong Rd, Neihu 114, Taipei, Taiwan; 2 Graduate Institute of Aerospace and Undersea Medicine, National Defense Medical Center, Taipei, Taiwan; 3 Division of Nephrology, Department of Medicine, Tri-Service General Hospital, National Defense Medical Center, Taipei, Taiwan; 4 Graduate Institute of Medical Sciences, National Defense Medical Center, Taipei, Taiwan

**Keywords:** STE20/SPS1-related proline/alanine-rich kinase, Hyperoxia, Alveolar epithelium, Claudin-18, p38 MAPK

## Abstract

**Background:**

Hyperoxia downregulates the tight junction (TJ) proteins of the alveolar epithelium and leads to barrier dysfunction. Previous study has showed that STE20/SPS1-related proline/alanine-rich kinase (SPAK) interferes with the intestinal barrier function in mice. The aim of the present study is to explore the association between SPAK and barrier function in the alveolar epithelium after hyperoxic exposure.

**Methods:**

Hyperoxic acute lung injury (HALI) was induced by exposing mice to > 99% oxygen for 64 h. The mice were randomly allotted into four groups comprising two control groups and two hyperoxic groups with and without SPAK knockout. Mouse alveolar MLE-12 cells were cultured in control and hyperoxic conditions with or without SPAK knockdown. Transepithelial electric resistance and transwell monolayer permeability were measured for each group. In-cell western assay was used to screen the possible mechanism of p-SPAK being induced by hyperoxia.

**Results:**

Compared with the control group, SPAK knockout mice had a lower protein level in the bronchoalveolar lavage fluid in HALI, which was correlated with a lower extent of TJ disruption according to transmission electron microscopy. Hyperoxia down-regulated claudin-18 in the alveolar epithelium, which was alleviated in SPAK knockout mice. In MLE-12 cells, hyperoxia up-regulated phosphorylated-SPAK by reactive oxygen species (ROS), which was inhibited by indomethacin. Compared with the control group, SPAK knockdown MLE-12 cells had higher transepithelial electrical resistance and lower transwell monolayer permeability after hyperoxic exposure. The expression of claudin-18 was suppressed by hyperoxia, and down-regulation of SPAK restored the expression of claudin-18. The process of SPAK suppressing the expression of claudin-18 and impairing the barrier function was mediated by p38 mitogen-activated protein kinase (MAPK).

**Conclusions:**

Hyperoxia up-regulates the SPAK-p38 MAPK signal pathway by ROS, which disrupts the TJ of the alveolar epithelium by suppressing the expression of claudin-18. The down-regulation of SPAK attenuates this process and protects the alveolar epithelium against the barrier dysfunction induced by hyperoxia.

## Background

Supplemental oxygen is used to counteract tissue hypoxemia, but breathing a high concentration of oxygen for a prolonged period may cause fatally hyperoxic acute lung injury (HALI) in both adults and premature infants [1–3]. HALI presents with histopathological changes that include increased microvascular permeability, the influx of protein-rich fluid, and the formation of lung edema, which are similar to the changes seen in the acute respiratory distress syndrome. In HALI, reactive oxygen species (ROS) are generated due to prolonged hyperoxia and destroy alveolar epithelial cells through both necrosis and apoptosis [4, 5]. Hyperoxia also results in the accumulation of inflammatory mediators within the lungs. This process involves protein kinases such as serine-threonine kinase Akt, mitogen-activated protein kinases (MAPK), protein kinase C, and transcription factors such as NF-E2-related transcription factor 2 and nuclear factor-κB [5–7].

In the alveolar epithelium, transmembrane and peripheral proteins compose the tight junction (TJ) that attach cells tightly to their neighbors and form a barrier. The change of TJ modulates the paracellular space and plays an important role in maintaining the hydration, ionic, and solute balance of alveoli [8, 9]. Three transmembrane protein families are found in the TJ: occludins, claudins, and junctional adhesion molecules [10, 11]. TJ proteins that are frequently expressed in the lungs include claudin-1, claudin-3, claudin-4, claudin-5, claudin-7, claudin-18, occludin, ZO-1, and ZO-2 [12–14]. Previous studies have shown that hyperoxia downregulates the expression of TJ proteins in the alveolar epithelium in HALI, which leads to barrier dysfunction [15–17].

STE20/SPS1-related proline/alanine-rich kinase (SPAK) is a member of the SPS1 subfamily of the mammalian STE20-related protein kinase family and is expressed ubiquitously throughout the body [18, 19]. In the alveolar epithelium, SPAK is a downstream substrate of WNK4 kinase and an upstream regulator of Na–K–Cl cotransporter (NKCC) [20, 21]. The phosphorylation of SPAK during osmotic stress regulates the activity of NKCC1 and maintains alveolar fluid homeostasis [22]. The role of the WNK4-SPAK-NKCC1 pathway involved in lung injury have been investigated previously. The mice with SPAK knockout exhibited longer survival than wild-type controls in HALI. In this study, SPAK may interfere with the course of lung injury by modulating alveolar fluid clearance via NKCC1 [21].

In addition to NKCC1, SPAK modulates other downstream effectors that are involved in the formation of epithelial barrier. In a model of intestinal inflammation, the production of inflammatory cytokines and aggravated bacterial translocation were facilitated in SPAK transgenic mice [23]. Another study showed that mice with SPAK deficiency had less proinflammatory cytokine production and luminal bacteria translocation [24]. In these two studies, changes in transepithelial resistance were observed [23, 24]. However, no study has explored the association between SPAK and the barrier function of the alveolar epithelium. The present study explores the role of SPAK in hyperoxia-induced alveolar barrier dysfunction both in vitro and in vivo.

## Methods

### Transgenic SPAK knockout mice

The mouse mutants for SPAK were provided by Dr Sung-Sen Yang (National Defense Medical Center, Taipei, Taiwan). SPAK+/– littermates were generated as described previously [20, 25]. SPAK+/– littermates were intercrossed to generate SPAK–/– (SPKA-KO) and wild-type (WT) mice. Approval for the project protocol was obtained from the Institutional Animal Care and Use Committee of the National Defense Medical Center. The mice were bred and maintained in pathogen-free animal facilities at the Laboratory Animal Center of the National Defense Medical Center (Taipei, Taiwan).

### Animal model of hyperoxic acute lung injury

The study was performed with 10 to 12-week-old male mice. The mice were randomly allotted into four groups comprising two control groups and two hyperoxic groups with and without SPAK knockout (n = 6 per group; totally 24 mice were used). We achieved random allocation by tossing a coin. In the control group, mice were kept in identical chambers and exposed to room air only (n = 6 per cage). In the hyperoxic group, mice were exposed to > 99% oxygen in an airtight chamber with a ventilation flow of 5 L/min for 64 h (n = 6 per cage). The CO_2_ concentration was kept at < 0.1%, and the temperature was controlled between 25 and 26 °C. In each cage, all mice were provided access to food and water ad libitum to minimise potential confounders such as the order of measurements and animal location.

At the end of the animal experiment, the mice were anaesthetized through intraperitoneal injection of Zoletil (Virbac, Carros, France; 35 mg/kg body weight) and Rompun (Bayer, Leverkusen, Germany; 10 mg/kg body weight). We performed a tracheostomy and a median sternotomy under general anesthesia. Then, the mice were euthanized by cardiac puncture before regaining consciousness. After euthanasia, bronchoalveolar lavage fluid (BALF) was obtained by lavaging the left lung twice with 0.5 mL of saline from the tracheostomy. The blood samples and right lung tissues were collected for further evaluation.

### Histopathology

The fixed and sectioned lung tissues were stained with eosin and hematoxylin. Using light microscopy, we analyzed the numbers of polymorphonuclear neutrophils and lung injury score of the lung tissue. We examined a minimum of 10 randomly selected fields for neutrophil infiltration in the airspace or vessel wall and the thickening of the alveolar wall. A four-point scale was used for scoring the lung damage as follows: none (0), mild (1), moderate (2), or severe (3). Two pathologists were blinded to score the experimental conditions. The lung injury score was summed up the two resulting scores.

### Bronchoalveolar lavage fluid protein

The BALF was centrifuged at 200×*g* for 10 min to remove cells and cellular debris. The protein concentrations were determined by a Pierce™ BCA protein assay kit (Thermo Fisher Scientific).

### Transmission electron microscopy

Ultrastructural characterization of the cytological alterations was performed by a following a procedure that is detailed in previous publication. Briefly, lung tissue blocks (maximal 1 mm^3^) were immediately dissected after euthanasia, kept overnight at 4 °C in fixative (4% paraformaldehyde and 2.5% glutaraldehyde in 1xPBS; pH 7.4), and postfixed in 1% OsO_4_ in the same buffer. After dehydration in graded ethanol the blocks were finally embedded in Spurr’s resin (Spurr Low Viscosity Embedding Kit; EMS ®). Semithin sections (0.5 μm thick) were cut with a glass knife on a Leica EM UC7 ultramicrotome and stained with toluidine blue. For TEM, ultrathin sections were cut on a Leica® Ultracut UC7 Ultramicrotome with a diamond knife. The sections were stained with uranyl acetate and lead citrate and examined with a FEI Tecnai G2 F20 S-TWIN Electron Microscope at 120 kV.

### MLE-12 cells and exposure to hyperoxia

MLE-12 cells, the type II mouse-lung epithelial cell, were purchased from ATCC (Manassas, VA). Cells were cultured in a 50:50 mixed medium of DMEM and Ham’s F-12 supplemented with 4% FBS, insulin (5 μg/mL), transferrin (10 μg/mL), sodium selenite (30 nM), hydrocortisone (10 nM), β-estradiol (10 nM), HEPES (10 nM), and L-glutamine (2 mM). In transgenic studies, MLE-12 cells were cultured on 6-well plates. At 60–75% confluence, transient transfection was carried out using SPAK siRNA (50 nM) (Dharmacon RNA Technologies) as the SPAK-knockdown (SPAK-KD) or siCONTROL Non-Targeting siRNA (50 nM) as the negative control. In the hyperoxic group, cells were placed in an incubator filled with 95% O_2_ and 5% CO_2_ at 37 °C for 48 h. In the control group, cells were kept in 21% O_2_ and 5% CO_2_ at 37 °C for 48 h.

### Transepithelial electric resistance measurements

Electric cell-substrate impedance sensing (ECIS) measurements were performed using 8W1E + electrode arrays on an ECIS Zθ instrument (Applied Biophysics, Troy, NY). The measurements were performed as described previously [26]. A baseline was established using culture medium (400 μL well^−1^). The resistance was recorded in units of Ω at a frequency of 500 Hz. At 48 h after transfection, the cells were sub-cultured on an ECIS array. Exposure to hyperoxia or normoxia began when the electrode was covered with a monolayer of cells. The ECIS allows for a sensitive determination of the amount of current passing between cells and the resistance of the barrier (*Rb*) (in units of Ω cm^2^). *Rb* is a robust reporter of barrier function.

### Transwell monolayer permeability assay

MLE-12 cells were grown as a monolayer in 6.5-mm-diameter transwell filter inserts with a pore size of 3.0 μm (Corning Life Sciences, Lowell, MA) to measure the paracellular permeability. After 48 h of exposure to hyperoxia, we replaced the medium of the upper chamber by medium containing albumin-fluorescein isothiocyanate (4 kDa, 2 mg/mL). Four hours later, 100-μL samples from the lower chambers were collected and analyzed for fluorescein isothiocyanate intensity using a fluorometric plate reader with an excitation of 494 nm and emission at 520 nm.

### Immunofluorescence staining

We performed immunofluorescence staining by a published procedure [27]. Lung sections were treated with primary rabbit polyclonal antibody, claudin-18 (diluted 1:200, Proteintech, IL, USA), and phosphorylated-SPAK (p-SPAK) (diluted 1:100, OriGene, MD, USA) for immunofluorescent labeling. The secondary antibody was goat anti-mouse IgG-FITC (diluted 1:200, Santa Cruz Biotechnology, USA) and Rhodamine (TRITC) AffiniPure Goat Anti-Rabbit IgG (diluted 1:200, Jackson ImmunoResearch Inc. PA, USA). The slides were mounted with VECTASHIELD Antifade Mounting Medium (Vector Laboratories, Inc. CA, USA) and DAPI. Images were obtained using a DeltaVision system (Applied Precision) comprising a wide-field inverted microscope (model IX-71; Olympus) with × 60/1.42 Plan Apo N or × 100/1.40 Super-Plan APO objectives.

### In-cell western assay

An in-cell western assay was performed using an Odyssey Infrared Imaging System (LICOR Biosciences, NE, USA). The cells were cultured at a density of 1.2 × 10^4^ cells/well in 96-well culture plates and incubated overnight in complete culture medium. At 70% confluence, the cells were pre-treated with ROS inhibitors for 30 min and then exposed to hyperoxia for 24 h. Cells were fixed with refrigerated 75% EtOH and stained with phosphorylated SPAK (Ser311) (diluted 1:200, OriGene, Rockville, MD) and beta-actin (diluted 1:200, Sigma Chemical Company, MO, USA) at 4 °C overnight. Anti-rabbit IRDye® 680RD-labeled (1:5000) and anti-mouse IRDye® 800-labeled CW (1:5000) antibodies (LICOR Biosciences, NE, USA) were used as secondary antibodies at room temperature for 1 h and were detected by the 700 and 800-nm channels, respectively.

### Western blot analysis

Specimens of cells and tissue were harvested and incubated for 10 min on ice with lysis buffer. The lysates were then centrifuged at 14,000 RCF for 10 min at 4 °C, and the supernatants were collected. Equal amounts of protein samples were separated using sodium dodecyl sulfate–polyacrylamide gel electrophoresis (SDS-PAGE), followed by transfer onto a polyvinylidene difluoride membrane (Millipore). Western blot analyses were performed with the relevant antibodies: claudin-18 (diluted 1:200, Thermo Fisher Scientific Inc, IL, USA), p-SPAK (diluted 1:1000, OriGene, MD, USA), phosphorylated-p38 (p-p38) (diluted 1:1000, Cell Signaling Technology, USA), total-p38 (T-p38) (diluted 1:1000, Cell Signaling Technology, USA), beta-actin (diluted 1:1000, Sigma Chemical Company, MO, USA) and GAPDH (diluted 1:1000, Thermo Fisher Scientific Inc, IL, USA).

### Real-time PCR

We isolated total RNA by an RNA-spin total RNA extraction kit (Intron Biotechnology, Korea) according to the manufacturer’s instructions. 2 µg of RNA was used to synthesize cDNA by a High-Capacity cDNA Archive Kit (Applied Biosystems, CA, USA). Using TaqMan assays (Applied Biosystems, CA, USA), quantitative real-time PCR was performed for claudin-18 (Mm00517322_m1) and GAPDH (Mm99999915_g1). Each sample was centrifuged and placed in a QuantStudio™ 5 Real-Time PCR System (Thermo Fisher Scientific, MA, USA), which was analyzed in triplicate on a 96-well plate. We used the following program to analyze: 2 min at 50 °C, 10 min at 95 °C, and 40 cycles of 15 s at 95 °C and 1 min at 60 °C, and used the 2^−ΔΔCT^ method to calculate the relative gene expression.

### Statistical analysis

All results are expressed as the mean ± standard deviation of the mean. There was no data point that was not included in the analysis. We used one-way analysis of covariance (ANOVA) to compare the differences between the study groups, and then Bonferroni's correction was employed for post-hoc comparisons. A *p* value < 0.05 was considered significant.

## Results

### SPAK knockout mice preserves barrier integrity in mice with hyperoxic acute lung injury

**Fig. 1:**
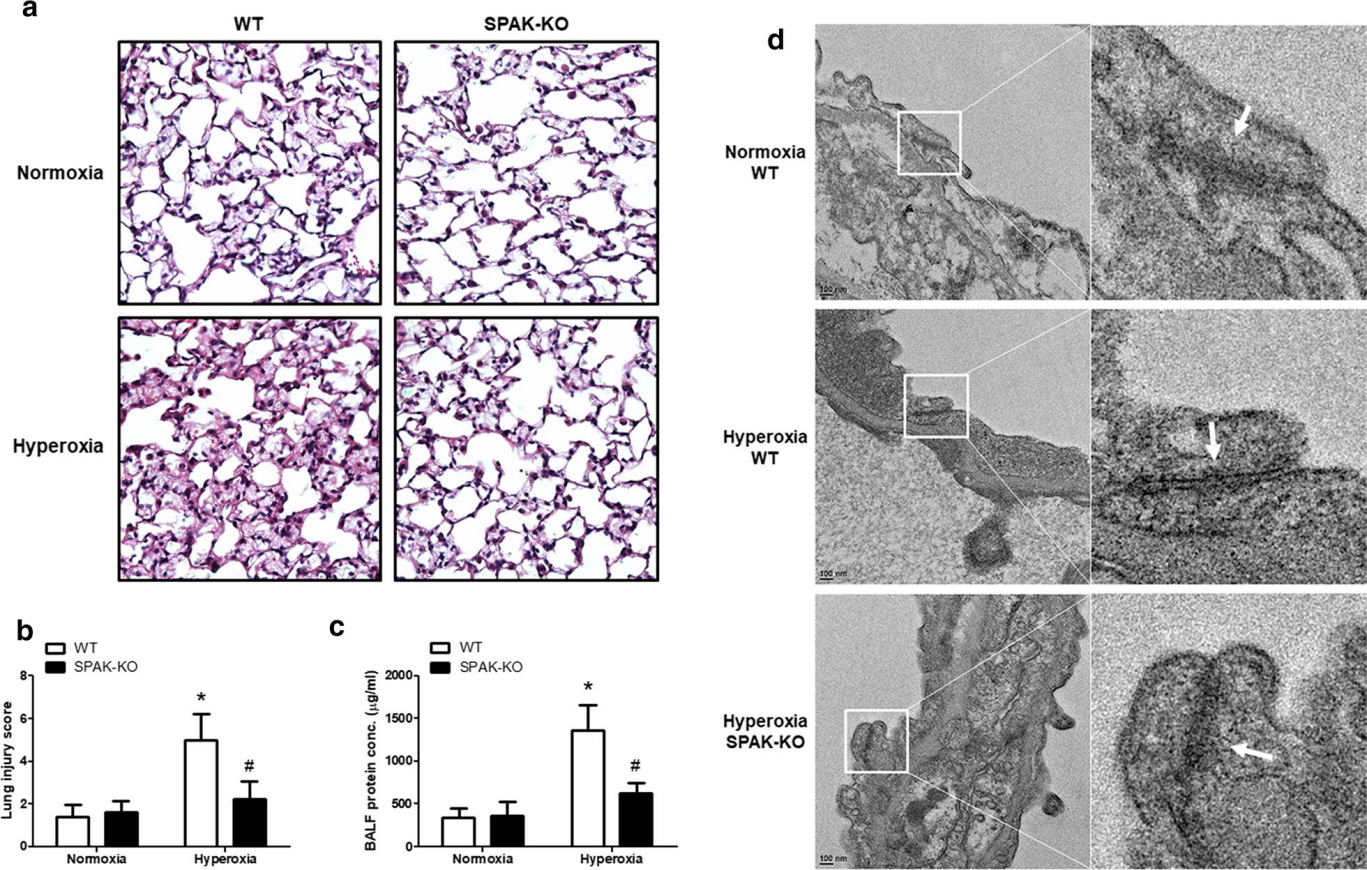
Effects of SPAK on the barrier integrity in hyperoxic acute lung injury. **a** Representative images of hematoxylin and eosin staining of mouse lungs (× 400 magnification). **b** Lung injury score by histological evaluation of mouse lung tissues (n = 6). **c** Protein concentration in bronchoalveolar lavage fluid (n = 6). **d** Transmission electron microscopy for the morphology of tight junctions in alveolar epithelial cells (arrows). *BALF* bronchoalveolar lavage fluid, *WT* wild type, *SPAK-KO* SPAK knock-out. Data are expressed as the means ± SD. *p < 0.05 versus normoxia WT; ^#^*p* < 0.05 versus hyperoxia WT

Histological evaluation of the lung tissues indicated a higher lung injury score after hyperoxic exposure for 64 h. Knockout SPAK significantly mitigated the increases in lung injury score (Fig. 1a, b). The protein concentration of BALF was measured as an indicator of dysfunction in the alveolar capillary barrier. After hyperoxia, the protein concentration increased significantly. SPAK-KO mice had significantly lower protein concentrations than WT mice (Fig. 1c). Transmission electron microscopy was used to evaluate the morphology of TJ in the alveolar epithelial cells of mice after hyperoxic exposure. In lung tissue from WT mice, the apical side indicated a loss of TJ and the presence of paracellular gaps between alveolar epithelial cells. Lung tissue from SPAK-KO mice showed a lesser extent of TJ disruption (Fig. 1d). The protein concentration of BALF was correlated with the extent of TJ disruption.

### Phosphorylation of SPAK mediates the decline in claudin-18 expression of the alveolar epithelium in mice with hyperoxic acute lung injury

**Fig. 2:**
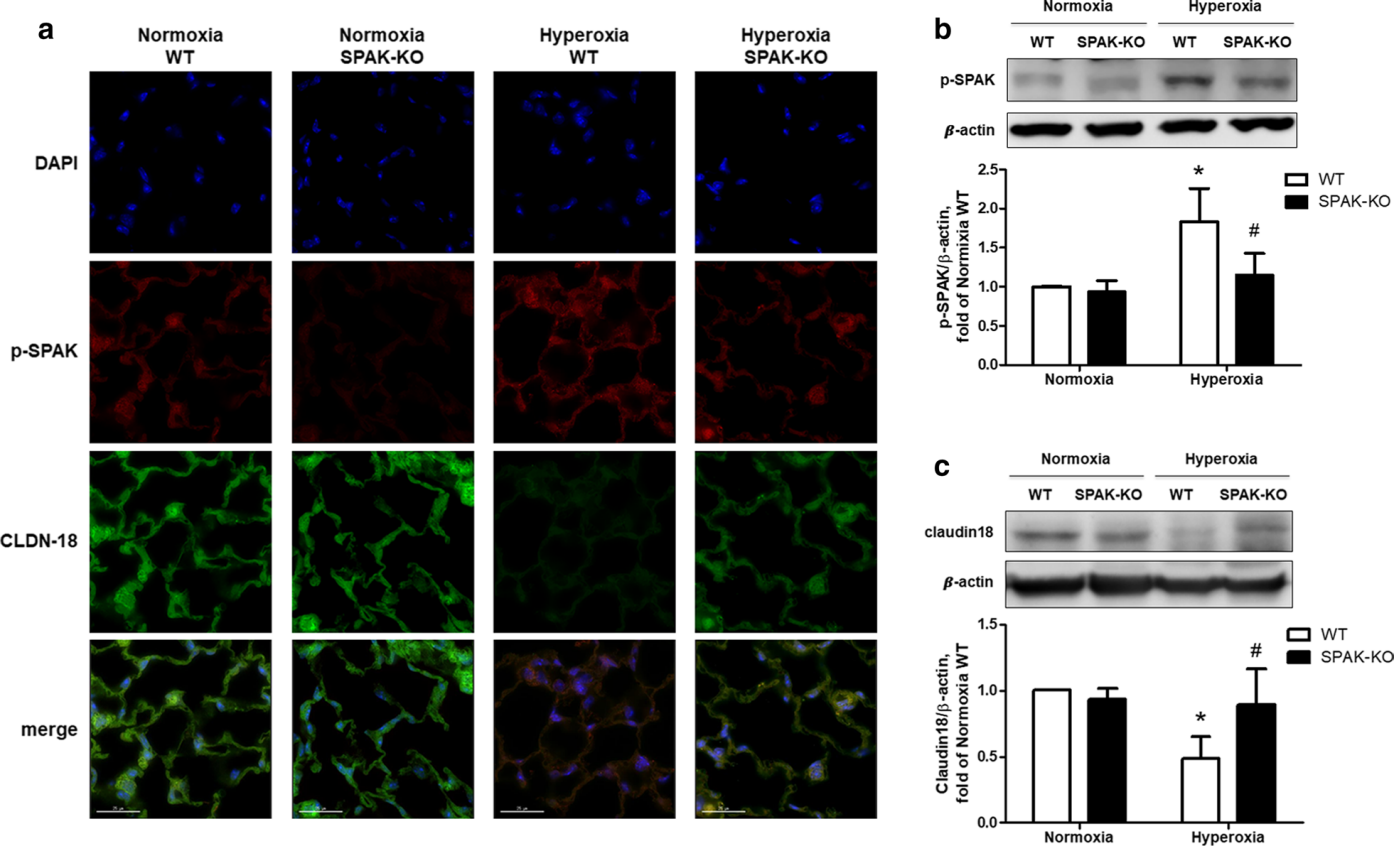
Effects of hyperoxia on the expressions of p-SPAK and claudin-18 of alveolar epithelium in hyperoxic acute lung injury. **a** Representative images of immunofluorescence staining for p-SPAK (TRITC-labeled red) and claudin-18 (FITC-labeled green) of mouse lungs (original magnification × 600). Nuclei were counterstained with DAPI (blue). **b** p-SPAK expressions in mouse lungs determined by western blot analysis (n = 5). **c** Claudin-18 expressions in mouse lungs determined by western blot analysis (n = 6). WT: wild type. SPAK-KO: SPAK knock-out. p-SPAK: phosphorylated-SPAK. Data are expressed as the means ± SD. *p < 0.05 versus normoxia WT; ^#^p < 0.05 versus hyperoxia WT

The p-SPAK and claudin-18 of alveolar epithelium were observed by lung tissue immunofluorescence double staining. Markedly increased p-SPAK in the alveolar epithelium of WT mice exposed to hyperoxia for 64 h was noted, and the increase was significantly less pronounced in the SPAK-KO mice. The expression of claudin-18 in the alveolar epithelium was significantly lower in the hyperoxic group than the normoxic group in WT mice. In SPAK-KO mice, the expression of claudin-18 was restored in the hyperoxic group (Fig. 2a). The effects of hyperoxia in activating p-SPAK and SPAK-KO in maintaining claudin-18 expression after hyperoxia were validated by western blot analysis for the lung tissues (Fig. 2b, c).

### Hyperoxia up-regulates phosphorylated-SPAK by ROS in MLE-12 cells

**Fig. 3:**
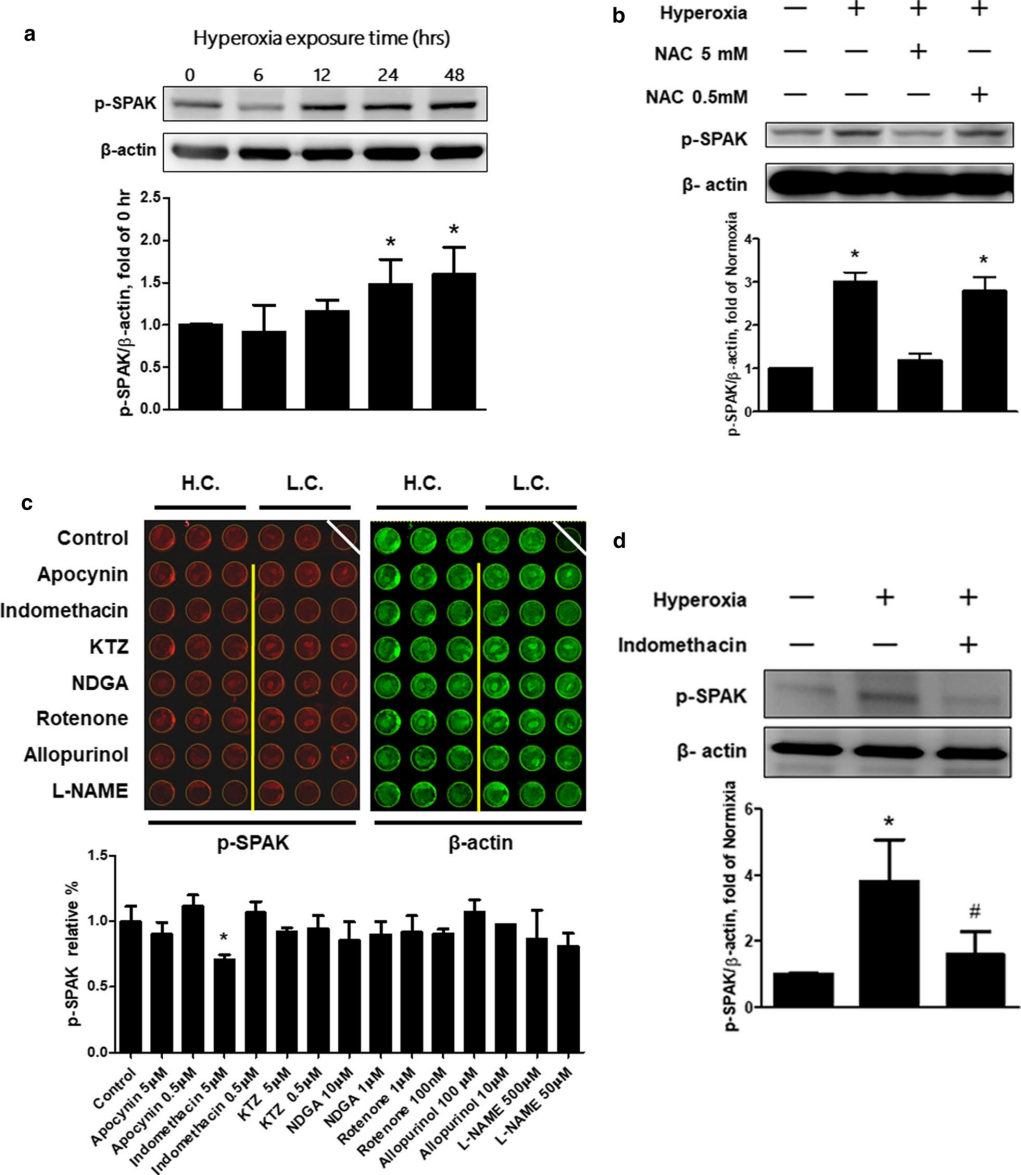
Effects of ROS on the expressions of p-SPAK in MLE-12 cells exposed to hyperoxia. **a** Effects of hyperoxia duration on p-SPAK expressions determined by western blot analysis (n = 4). *p < 0.05 versus 0 h. **b** Effects of n-acetylcysteine pretreatment on p-SPAK expressions in MLE-12 cells exposed to hyperoxia determined by western blot analysis (n = 3). *p < 0.05 versus normoxia control; ^#^*p* < 0.05 versus hyperoxia control. **c** Effects of ROS-generating enzyme inhibitors on p-SPAK expressions detected by in-cell western assay and the quantitative values from intensity of p-SPAK (n = 3). *p < 0.05 versus control. **d** Effects of indomethacin 5 μM on p-SPAK expressions determined by western blot analysis (n = 3). *p < 0.05 versus normoxia control; ^#^*p* < 0.05 versus hyperoxia control. *WT* wild type, *NAC* n-acetylcysteine, *H.C.* high concentration, *L.C.* low concentration, *p-SPAK* phosphorylated-SPAK. Data are expressed as the mean ± SD

MLE-12 cells were treated with hyperoxia to assess the change of p-SPAK in the alveolar epithelium. The p-SPAK was significantly activated after exposure to hyperoxia for 24 h (Fig. 3a). To assess the effects of ROS in activating p-SPAK, MLE-12 cells were pretreated with n-acetylcysteine (NAC) and then treated with hyperoxia. The p-SPAK was activated by hyperoxia, and pretreatment with a NAC concentration of 5 mM suppressed this activation (Fig. 3b). To screen the possible mechanism of p-SPAK being induced by ROS, an in-cell western assay was used for MLE-12 cells exposed to 24 h of hyperoxia. ROS-generating enzyme inhibitors were applied as pretreatment before hyperoxia, including apocynin, indomethacin, ketoconazole, NADG, rotenone, allopurinol, and L-NAME. The activation of p-SPAK was not suppressed by low concentrations of ROS-generating enzyme inhibitors, but it was significantly suppressed by a high concentration of indomethacin (Fig. 3c). Western blot analysis validated the effect of indomethacin in hyperoxia-induced p-SPAK activation (Fig. 3d).

### SPAK mediates the decline in claudin-18 expression and barrier function of MLE-12 cells after hyperoxic exposure

**Fig. 4:**
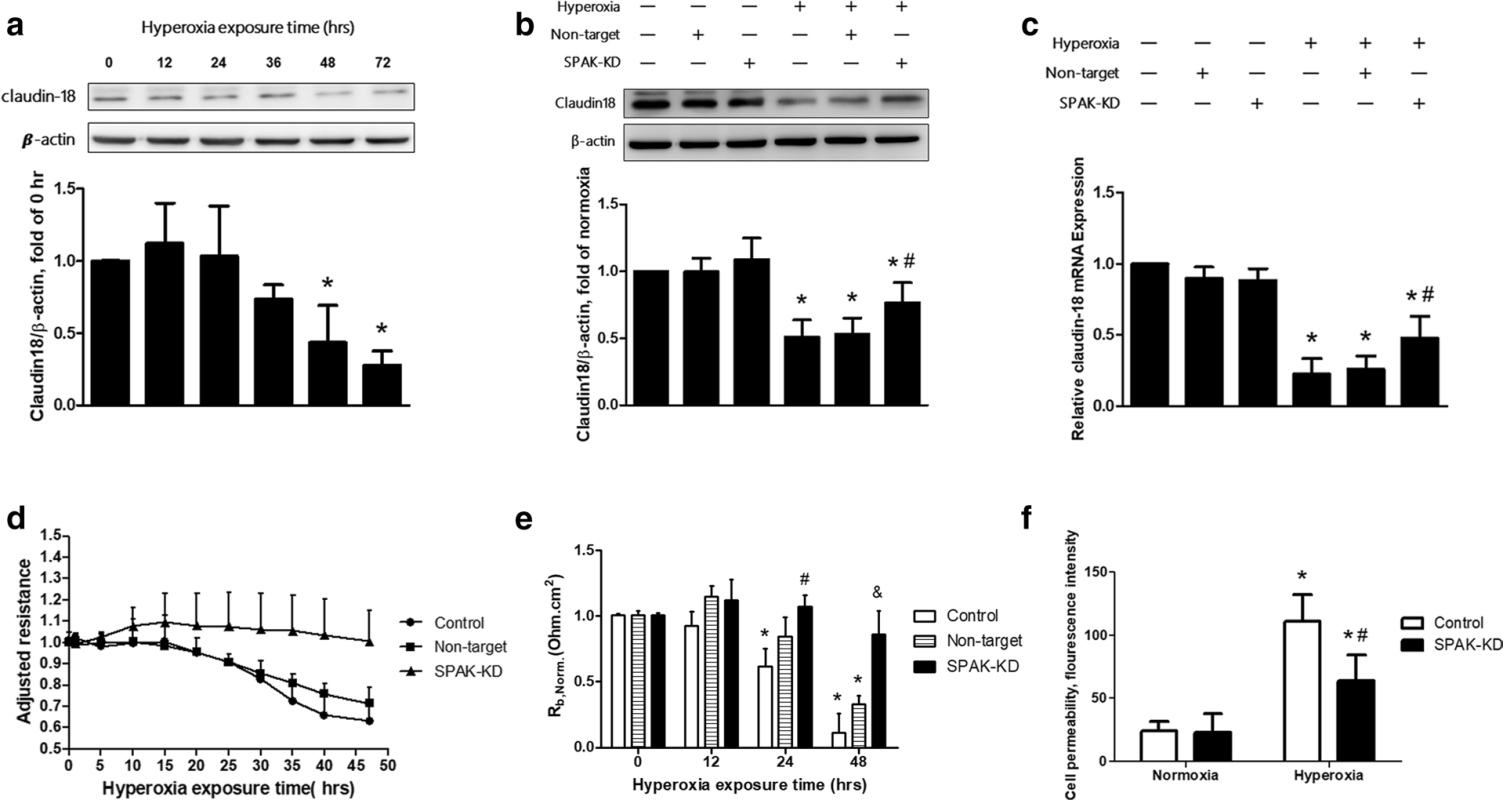
Effects of p-SPAK on the expressions of claudin-18 and barrier function in MLE-12 cells exposed to hyperoxia. **a** Effects of exposure duration on expressions of claudin-18 in MLE-12 cells determined by western blot analysis (n = 4). *p < 0.05 versus 0 h. **b**, **c** Effects of SPAK-KD on expressions of claudin-18 in MLE-12 cells determined by western blot analysis and mRNA levels (n = 4). *p < 0.05 versus normoxia control; ^#^*p* < 0.05 versus hyperoxia control. **d** Transepithelial electrical resistance of MLE-12 cells measured by ECIS (n = 4). **e** Barrier resistance (*Rb*) of MLE-12 cells measured by ECIS (n = 4). *p < 0.05 versus 0 h control; ^#^*p* < 0.05 versus 24 h control; ^&^*p* < 0.05 versus 48 h control. **f** Paracellular permeability assay using MLE-12 cells cultured with FITC-dextran (n = 5). *p < 0.05 versus normoxia control; ^#^*p* < 0.05 versus hyperoxia control. *SPAK-KD* SPAK knock-down. Data are expressed as the means ± SD

We measured the expression of claudin-18 in MLE-12 cells and found that hyperoxia suppressed the expression in a time-dependent manner. A significant decrease was noted after 48 h of exposure (Fig. 4a). We performed immunoblotting and quantitative real-time PCR to demonstrate the effect of SPAK on the expression of claudin-18 in MLE-12 cells exposed to hyperoxia for 48 h. The claudin-18 expression was significantly decreased in the control group in comparison to the SPAK-KD group (Fig. 4b, c). We measured the transepithelial electrical resistance of the monolayer of MLE-12 cells by ECIS to assess the effect of SPAK on the barrier integrity of the alveolar epithelium. A lower resistance (at a frequency of 500 Hz in ECIS measurements) was observed in the control group. The decrease of resistance caused by hyperoxic exposure was restored in the SPAK-KD group (Fig. 4d). The *Rb* decreased significantly after hyperoxic exposure in the control group, but not in the SPAK-KD group (Fig. 4e). In addition, hyperoxic exposure resulted in an increased paracellular permeability, which was alleviated by knocking down SPAK (Fig. 4f). These findings suggest that down-regulating SPAK attenuates the hyperoxia-induced barrier dysfunction in the alveolar epithelium.

### SPAK modulates hyperoxia-induced barrier dysfunction and suppression of claudin-18 via p38 MAPK in MLE-12 cells

**Fig. 5:**
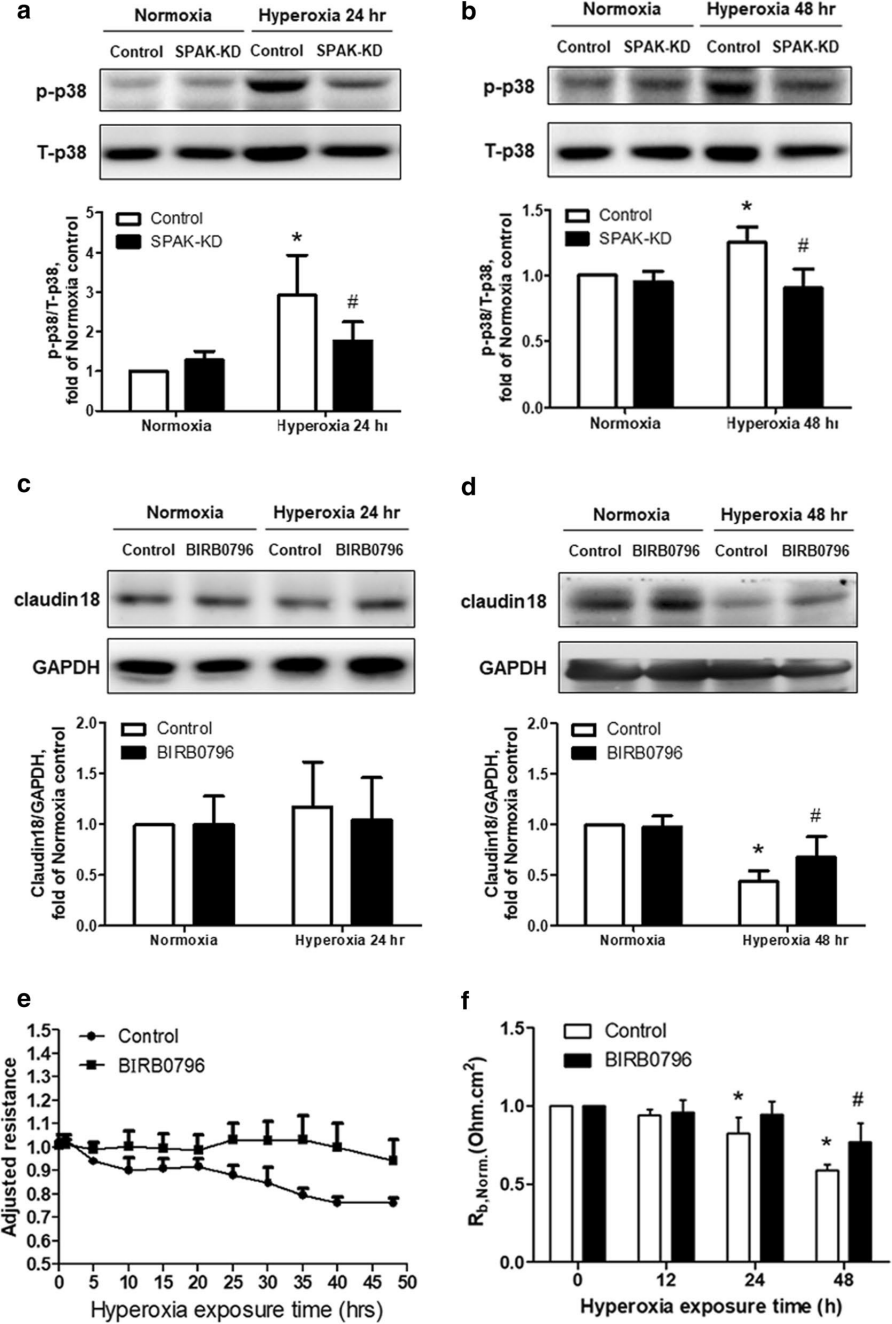
Effects of p38 MAPK on the expressions of claudin-18 and barrier function in MLE-12 cells after hyperoxic exposure. **a**, **b** Expressions of total p38 MAPK and phosphor brylated p38 MAP K after hyperoxic exposure for 24 h (n = 6) and 48 h (n = 6). *p < 0.05 versus normoxia control; ^#^*p* < 0.05 versus hyperoxia control. **c**, **d** Claudin-18 expressions after treatment by p38 MAPK inhibitor BIRB-796 followed by exposure to hyperoxia for 24 h (n = 6) and 48 h (n = 5). *p < 0.05 versus normoxia control; ^#^p < 0.05 versus hyperoxia control. **e** Transepithelial electrical resistance treated by BIRB-796 and measured by ECIS (n = 4). **f** Barrier resistance (*Rb*) of MLE-12 cells measured by ECIS (n = 4). *p < 0.05 versus 0 h control; ^#^*p* < 0.05 versus 48 h control. *SPAK-KD* SPAK knock-down, *T-p38* total-p38, *p-p38* phosphorylated-p38. Data are expressed as the mean ± SD

A previous study showed that SPAK acts as a mediator of stress-activated signals that activates p38 MAPK [28]. We performed immunoblotting of T-p38 and p-p38 expressions in MLE-12 cells exposed to hyperoxia for 24 and 48 h (Fig. 5a, b). The expression of p-p38 was activated by hyperoxia in the control group. In the SPAK-KD group, the expression decreased significantly. The role of p38 MAPK in modulating the expression of claudin-18 was assessed by pretreating MLE-12 cells with 2 µM of p38 MAPK inhibitor (BIRB-796), followed by hyperoxia for 48 h. Hyperoxia resulted in lower claudin-18 expression, which was restored by BIRB0796 (Fig. 5c, d). ECIS showed that pretreatment with p38 MAPK inhibitor restored the decrease of resistance in MLE-12 cells after hyperoxic exposure (Fig. 5e). The *Rb* decreased significantly in the control group after hyperoxic exposure, but not in the p38 MAPK inhibitor group (Fig. 5f). These findings indicate that SPAK modulates hyperoxia-induced barrier dysfunction and the suppression of claudin-18 via p38 MAPK in the alveolar epithelium.

## Discussion

**Fig. 6:**
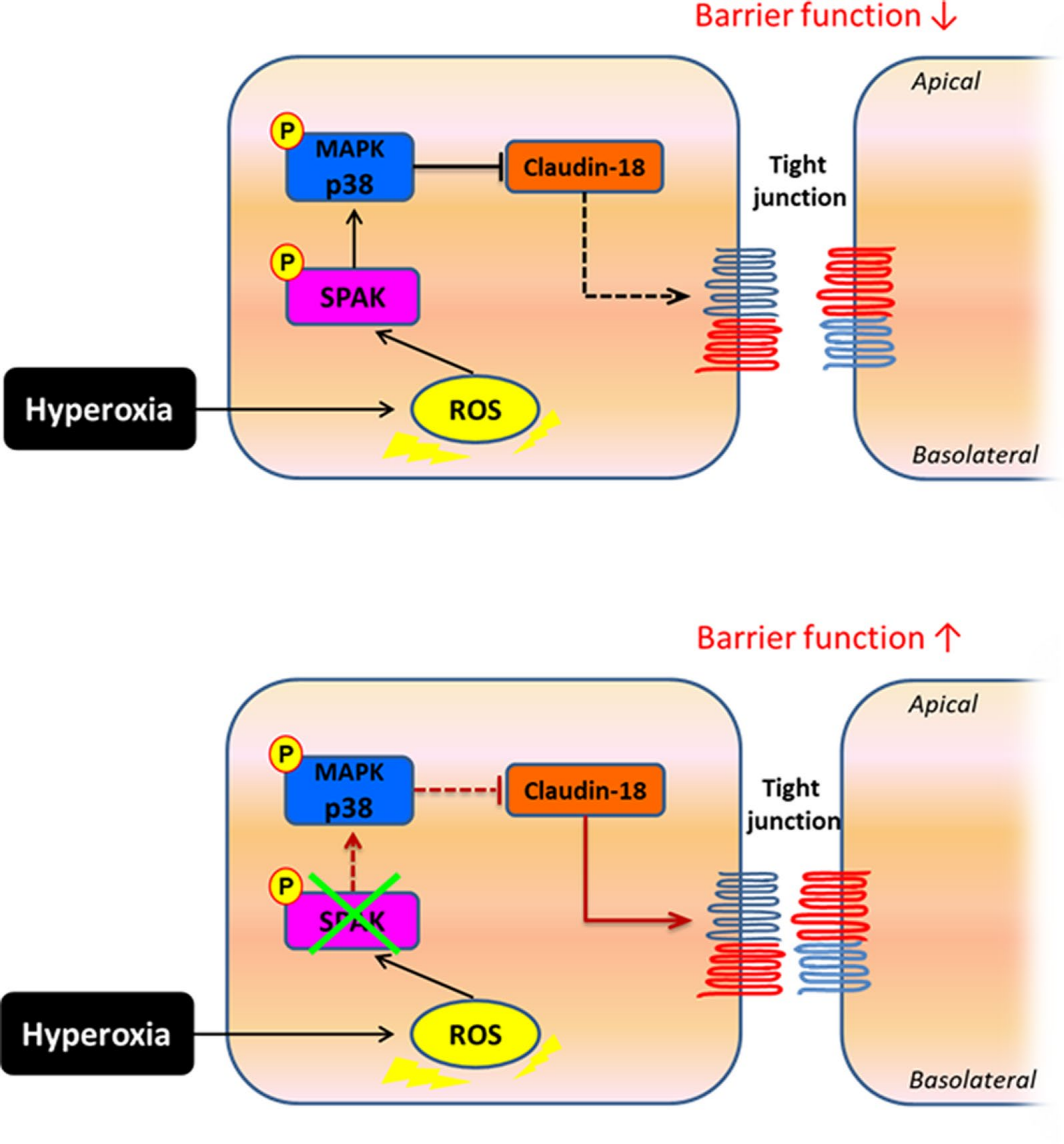
The mechanisms of SPAK-p38 MAPK signal pathway modulating alveolar barrier function after hyperoxic exposure. Hyperoxia up-regulates p-SPAK and actives p-p38 by ROS, which mediates the decline in claudin-18 expression and disrupts the TJ of the alveolar epithelium. Down-regulation of SPAK inhibits p-p38 and restores the expression of claudin-18, which preserves barrier integrity of the alveolar epithelium. *ROS* reactive oxygen species, *p-SPAK* phosphorylated-SPAK, *p-p38* phosphorylated-p38, *TJ* tight junction

We have presented the first research exploring the association between SPAK and barrier function of alveolar epithelium when exposed to hyperoxia both in vivo and in vitro. Hyperoxia up-regulates p-SPAK by ROS, which activates p-p38 and disrupts the TJ of the alveolar epithelium by suppressing the expression of claudin-18. Down-regulation of SPAK alleviates the phosphorylation of p38 and restores the expression of claudin-18, which protects the alveolar epithelium against the barrier dysfunction in HALI (Fig. 6).

The present study showed that hyperoxia activated p-SPAK of the alveolar epithelium by ROS. ROS is a family of active molecules containing free radicals that are involved in the injury of various cellular constituents, such as lipids, proteins, and DNA [29]. For epithelial cells, ROS disrupts the TJ directly by protein modifications such as thiol oxidation, phosphorylation, nitration, and carbonylation. Multiple inflammatory pathways may also be stimulated to impair the TJ and barrier function [14, 30, 31]. The known ROS-producing enzymes in mammalian cells are NADPH oxidases, xanthine oxidase, lipoxygenases, and cytochrome P450 [32]. ROS activates cyclooxygenase, and the activation of cyclooxygenase/prostaglandin synthase pathways may induce further ROS production through effects on different ROS-generating enzymes [33]. We found that p-SPAK activation induced by ROS was significantly inhibited by indomethacin, a cyclooxygenase inhibitor, suggesting that cyclooxygenase may play an essential role in activating p-SPAK in HALI.

The up-regulation of p-SPAK was correlated with the down-regulation of claudin-18 after hyperoxic exposure in this study. Claudin-18 is the claudin that is overwhelmingly expressed by alveolar epithelial cells [34]. In a bleomycin-induced experimental model of lung injury, the expression of claudin-18 and the integrity of the epithelial TJ were disturbed in the fibrotic lesions [35]. Claudin-18 may play an important role in the regulation of ion selectivity to mediate barrier function. A previous study has shown that claudin-18 knockout mice exhibit greater solute permeability than WT control mice. In monolayers formed by claudin-18-deficient alveolar epithelial cells, enlarged paracellular gaps were correlated with changes in the actin cytoskeleton [36]. Therefore, claudin-18 may be a crucial target modulated by SPAK in the alveolar epithelium after hyperoxic exposure.

In a mouse model of intestinal inflammation, SPAK altered the permeability of epithelial cells by regulating the expression of TJ proteins. SPAK-KO mice showed increased barrier function of the intestinal epithelium induced by dextran sulfate sodium. The changes of barrier function were associated with increased expressions of occludin, E-cadherin, β-catenin, and claudin-5, but there were no noticeable changes in the expressions of claudin-1, claudin-4, ZO-1, and ZO-2 [24]. Claudin-18 was not affected in this study, and we suppose that this may have been caused by the differences in the injury model and injured tissue. Further studies are warranted to identify the associations between SPAK and changes in other TJ proteins induced by hyperoxia.

Many factors contribute to the biological interactions with SPAK. SPAK functions as an upstream kinase of NKCC1, but previous studies have not shown associations between NKCC1 and epithelial barrier function. SPAK has several binding partners and effectors, such as apoptosis-associated tyrosine kinase, protein kinase C isotypes, glycoprotein CD46, heat shock protein, otoferlin, gelsolin, calcium binding protein, p21-activated protein kinase, and MAPKs [37]. Among these kinases, activation of p38 MAPK was found in mice with LPS-induced acute lung injury, and inhibition of p38 MAPK activity attenuated pulmonary edema formation and hyperpermeability [38]. A previous study has shown that ROS contributes to the activation of p38 MAPK, which was associated with microtubule destabilization and the formation of paracellular gaps in the vascular endothelium in mouse lungs [34]. In the present study, the expression of epithelial claudin-18 was also modulated by p38 MAPK when exposed to hyperoxia, which points out the multiple roles of p38 MPAK in barrier dysfunction induced by oxidative stress.

The pharmacological targeting of SPAK includes antagonizing the SPAK-WNK interaction, inhibiting SPAK activity, or disrupting SPAK activation by interfering with its binding to Μ025α/β. These approaches could be useful for regulating the homeostasis of the renal epithelium and may have therapeutic potential for NaCl-sensitive hypertension [39]. In addition to the role in blood pressure regulation, abrogating SPAK activity may be a therapeutic strategy for other diseases. The inactivation of SPAK was shown to reduce body weight gain in mice fed a high-fat diet, which occurred through the improvement of energy expenditure and insulin sensitivity [40]. The deletion of the WNK3-SPAK kinase complex in mice improves radiographic and clinical outcomes in malignant cerebral edema after ischemic stroke [41]. SPAK also plays a pathogenic role in IgA nephropathy through the activation of the NF-κB/MAPKs signaling pathway [42]. In endotoxemic mice, the deletion of SPAK not only reduces the elevation of nitric oxide levels but also improves vascular hyporeactivity to serotonin and phenylephrine [43]. The cellular model in the present study has shown the direct effect of the SPAK-p38 MAPK signal pathway on the alveolar epithelium after hyperoxic exposure. Although further human studies are warranted, our findings suggest that the selective inhibition of alveolar SPAK phosphorylation may yield a new opportunity to treat barrier dysfunction in HALI.

The main limitation of this study is the complex pathogenic condition of HALI. In the present study, we found that SPAK activated p-p38 in MLE-12 cells exposed to hyperoxia. However, hyperoxia triggers diverse effects that involve not only the alveolar epithelium and the pulmonary vascular endothelium, but also the inflammatory cells such as macrophages and neutrophils [8]. Although the cellular model in the present study demonstrates a direct benefit of SPAK-KD on the alveolar epithelium exposed to hyperoxia, the effects of SPAK on other cells remain uncertain. Further studies are warranted to identify the roles of SPAK in the crosstalk between epithelium, endothelium, and inflammatory cells in HALI.

## Conclusions

Hyperoxia up-regulates the SPAK-p38 MAPK signal pathway through ROS, which disrupts the TJ of the alveolar epithelium by suppressing the expression of claudin-18. The down-regulation of SPAK attenuates this process and protects the alveolar epithelium against the barrier dysfunction in HALI. The selective inhibition of SPAK phosphorylation in the alveolar epithelium may be a potential strategy for alleviating hyperoxia-induced barrier dysfunction.

## Data Availability

The data generated during this study are included in this published article.

## Abbreviations

TJ: Tight junction
SPAK: STE20/SPS1-related proline/alanine-rich kinase
HALI: Hyperoxic acute lung injury
ROS: Reactive oxygen species
MAPK: Mitogen-activated protein kinase
NKCC1: Na–K–Cl cotransporter 1
WT: Wild-type
LPS: Lipopolysaccharide
SPAK-KO: SPAK knockout
BALF: Bronchoalveolar lavage fluid
ECIS: Electric cell-substrate impedance sensing
p-SPAK: Phosphorylated-SPAK
p-p38: Phosphorylated-p38
T-p38: Total-p38
NAC: N-acetylcysteine
SPAK-KD: SPAK-knockdown

## References

[1] Helmerhorst HJF, Roos-Blom M-J, van Westerloo DJ, de Jonge E (2015). Association between arterial hyperoxia and outcome in subsets of critical illness: a systematic review, meta-analysis, and meta-regression of cohort studies*. Crit Care Med.

[2] Ni Y-N, Wang Y-M, Liang B-M, Liang Z-A (2019). The effect of hyperoxia on mortality in critically ill patients: a systematic review and meta analysis. BMC Pulm Med.

[3] Perez M, Robbins ME, Revhaug C, Saugstad OD (2019). Oxygen radical disease in the newborn, revisited: oxidative stress and disease in the newborn period. Free Radical Biol Med.

[4] Dias-Freitas F, Metelo-Coimbra C, Roncon-Albuquerque R (2016). Molecular mechanisms underlying hyperoxia acute lung injury. Respir Med.

[5] Porzionato A, Sfriso MM, Mazzatenta A, Macchi V, De Caro R, Di Giulio C (2015). Effects of hyperoxic exposure on signal transduction pathways in the lung. Respir Physiol Neurobiol.

[6] Gore A, Muralidhar M, Espey MG, Degenhardt K, Mantell LL (2010). Hyperoxia sensing: from molecular mechanisms to significance in disease. J Immunotoxicol.

[7] Kallet RH, Matthay MA (2013). Hyperoxic acute lung injury. Respir Care.

[8] Herrero R, Sanchez G, Lorente JA (2018). New insights into the mechanisms of pulmonary edema in acute lung injury. Ann Transl Med.

[9] Wittekindt OH (2017). Tight junctions in pulmonary epithelia during lung inflammation. Pflugers Arch.

[10] Blasig IE, Haseloff RF (2011). Tight junctions and tissue barriers. Antioxid Redox Signal.

[11] Van Itallie CM, Anderson JM (2018). Phosphorylation of tight junction transmembrane proteins: many sites, much to do. Tissue Barriers.

[12] Overgaard CE, Mitchell LA, Koval M (2012). Roles for claudins in alveolar epithelial barrier function. Ann N Y Acad Sci.

[13] Frank JA (2012). Claudins and alveolar epithelial barrier function in the lung. Ann N Y Acad Sci.

[14] Blasig IE, Bellmann C, Cording J, Del Vecchio G, Zwanziger D, Huber O, Haseloff RF (2011). Occludin protein family: oxidative stress and reducing conditions. Antioxid Redox Signal.

[15] Xu S, Xue X, You K, Fu J (2016). Caveolin-1 regulates the expression of tight junction proteins during hyperoxia-induced pulmonary epithelial barrier breakdown. Respir Res.

[16] Vyas-Read S, Vance RJ, Wang W, Colvocoresses-Dodds J, Brown LA, Koval M (2018). Hyperoxia induces paracellular leak and alters claudin expression by neonatal alveolar epithelial cells. Pediatr Pulmonol.

[17] Al-Shmgani HS, Moate RM, Macnaughton PD, Sneyd JR, Moody AJ (2013). Effects of hyperoxia on the permeability of 16HBE14o-cell monolayers—the protective role of antioxidant vitamins E and C. FEBS J.

[18] Murillo-de-Ozores AR, Chávez-Canales M, de Los Heros P, Gamba G, Castañeda-Bueno M. Physiological processes modulated by the chloride-sensitive WNK-SPAK/OSR1 kinase signaling pathway and the cation-coupled chloride cotransporters. Front Physiol. 2020;11:585907. 10.3389/fphys.2020.585907 PMC7606576 33192599

[19] Shekarabi M, Zhang J, Khanna AR, Ellison DH, Delpire E, Kahle KT (2017). WNK kinase signaling in ion homeostasis and human disease. Cell Metab.

[20] Lan CC, Peng CK, Tang SE, Lin HJ, Yang SS, Wu CP, Huang KL (2017). Inhibition of Na-K-Cl cotransporter isoform 1 reduces lung injury induced by ischemia-reperfusion. J Thoracic Cardiovasc Surg.

[21] Lin H-J, Wu C-P, Peng C-K, Lin S-H, Uchida S, Yang S-S, Huang K-L (2015). With-no-lysine kinase 4 mediates alveolar fluid regulation in hyperoxia-induced lung injury*. Crit Care Med.

[22] Brown A, Meor Azlan NF, Wu Z, Zhang J. WNK-SPAK/OSR1-NCC kinase signaling pathway as a novel target for the treatment of salt-sensitive hypertension. Acta Pharmacol Sin. 2020. 10.1038/s41401-020-0474-7. 10.1038/s41401-020-0474-7 PMC8115323 32724175

[23] Yan Y, Laroui H, Ingersoll SA, Ayyadurai S, Charania M, Yang S, Dalmasso G, Obertone TS, Nguyen H, Sitaraman SV (2011). Over-expression of Ste20-related proline/alanine rich kinase (SPAK) exacerbates experimental colitis in mice. J Immunol (Baltimore, Md: 1950).

[24] Zhang Y, Viennois E, Xiao B, Baker MT, Yang S, Okoro I, Yan Y (2013). Knockout of Ste20-like proline/alanine-rich kinase (SPAK) attenuates intestinal inflammation in mice. Am J Pathol.

[25] Yang S-S, Lo Y-F, Wu C-C, Lin S-W, Yeh C-J, Chu P, Sytwu H-K, Uchida S, Sasaki S, Lin S-H (2010). SPAK-knockout mice manifest Gitelman syndrome and impaired vasoconstriction. J Am Soc Nephrol.

[26] Szulcek R, Bogaard HJ, van Nieuw Amerongen GP (2014). Electric cell-substrate impedance sensing for the quantification of endothelial proliferation, barrier function, and motility. J Vis Exp.

[27] Shen C-H, Lin J-Y, Chang Y-L, Wu S-Y, Peng C-K, Wu C-P, Huang K-L (2018). Inhibition of NKCC1 modulates alveolar fluid clearance and inflammation in ischemia-reperfusion lung injury via TRAF6-mediated pathways. Front Immunol.

[28] Johnston AM, Naselli G, Gonez LJ, Martin RM, Harrison LC, DeAizpurua HJ (2000). SPAK, a STE20/SPS1-related kinase that activates the p38 pathway. Oncogene.

[29] Zhang J, Wang X, Vikash V, Ye Q, Wu D, Liu Y, Dong W (2016). ROS and ROS-mediated cellular signaling. Oxidative Med Cell Longev.

[30] Overgaard CE, Daugherty BL, Mitchell LA, Koval M (2011). Claudins: control of barrier function and regulation in response to oxidant stress. Antioxid Redox Signal.

[31] Sokolowska M, Quesniaux VFJ, Akdis CA, Chung KF, Ryffel B, Togbe D (2019). Acute respiratory barrier disruption by ozone exposure in mice. Front Immunol.

[32] Diebold L, Chandel NS (2016). Mitochondrial ROS regulation of proliferating cells. Free Radical Biol Med.

[33] Hernanz R, Briones AM, Salaices M, Alonso MJ (2014). New roles for old pathways? A circuitous relationship between reactive oxygen species and cyclo-oxygenase in hypertension. Clin Sci (London, England: 1979).

[34] Schlingmann B, Molina SA, Koval M (2015). Claudins: Gatekeepers of lung epithelial function. Semin Cell Dev Biol.

[35] Ohta H, Chiba S, Ebina M, Furuse M, Nukiwa T (2011). Altered expression of tight junction molecules in alveolar septa in lung injury and fibrosis. Am J Physiol-Lung Cell Mol Physiol.

[36] Li G, Flodby P, Luo J, Kage H, Sipos A, Gao D, Ji Y, Beard LL, Marconett CN, DeMaio L (2014). Knockout mice reveal key roles for claudin 18 in alveolar barrier properties and fluid homeostasis. Am J Respir Cell Mol Biol.

[37] Chen W, Yazicioglu M, Cobb MH (2004). Characterization of OSR1, a member of the mammalian Ste20p/germinal center kinase subfamily. J Biol Chem.

[38] Wang W, Weng J, Yu L, Huang Q, Jiang Y, Guo X (2018). Role of TLR4-p38 MAPK-Hsp27 signal pathway in LPS-induced pulmonary epithelial hyperpermeability. BMC Pulm Med.

[39] Zhang J, Karimy JK, Delpire E, Kahle KT (2017). Pharmacological targeting of SPAK kinase in disorders of impaired epithelial transport. Expert Opin Ther Targets.

[40] Torre-Villalvazo I, Cervantes-Pérez LG, Noriega LG, Jiménez JV, Uribe N, Chávez-Canales M, Tovar-Palacio C, Marfil-Garza BA, Torres N, Bobadilla NA (2017). Inactivation of SPAK kinase reduces body weight gain in mice fed a high-fat diet by improving energy expenditure and insulin sensitivity. Am J Physiol Endocrinol Metab.

[41] Zhao H, Nepomuceno R, Gao X, Foley LM, Wang S, Begum G, Zhu W, Pigott VM, Falgoust LM, Kahle KT (2017). Deletion of the WNK3-SPAK kinase complex in mice improves radiographic and clinical outcomes in malignant cerebral edema after ischemic stroke. J Cereb Blood Flow Metab.

[42] Lin T-J, Yang S-S, Hua K-F, Tsai Y-L, Lin S-H, Ka S-M (2016). SPAK plays a pathogenic role in IgA nephropathy through the activation of NF-OєB/MAPKs signaling pathway. Free Radical Biol Med.

[43] Shih C-C, Hsu L-P, Liao M-H, Yang S-S, Ho S-T, Wu C-C (2017). Effects of SPAK on vascular reactivity and nitric oxide production in endotoxemic mice. Eur J Pharmacol.

